# Time-Varying Network Models for the Temporal Dynamics of Depressive Symptomatology in Patients With Depressive Disorders: Secondary Analysis of Longitudinal Observational Data

**DOI:** 10.2196/50136

**Published:** 2024-04-18

**Authors:** Björn Sebastian Siepe, Christian Sander, Martin Schultze, Andreas Kliem, Sascha Ludwig, Ulrich Hegerl, Hanna Reich

**Affiliations:** 1 Psychological Methods Lab Department of Psychology University of Marburg Marburg Germany; 2 German Depression Foundation Leipzig Germany; 3 Department of Psychiatry and Psychotherapy University of Leipzig Medical Center Leipzig Germany; 4 Department of Psychology Goethe University Frankfurt Germany; 5 Adesso SE Dortmund Germany; 6 Institute for Applied Informatics University Leipzig Leipzig Germany; 7 Department for Psychiatry, Psychosomatics and Psychotherapy Goethe University Frankfurt Germany; 8 Depression Research Center of the German Depression Foundation Department for Psychiatry, Psychosomatics and Psychotherapy Goethe University Frankfurt Germany

**Keywords:** depression, time series analysis, network analysis, experience sampling, idiography, time varying, mobile phone

## Abstract

**Background:**

As depression is highly heterogenous, an increasing number of studies investigate person-specific associations of depressive symptoms in longitudinal data. However, most studies in this area of research conceptualize symptom interrelations to be static and time invariant, which may lead to important temporal features of the disorder being missed.

**Objective:**

To reveal the dynamic nature of depression, we aimed to use a recently developed technique to investigate whether and how associations among depressive symptoms change over time.

**Methods:**

Using daily data (mean length 274, SD 82 d) of 20 participants with depression, we modeled idiographic associations among depressive symptoms, rumination, sleep, and quantity and quality of social contacts as dynamic networks using time-varying vector autoregressive models.

**Results:**

The resulting models showed marked interindividual and intraindividual differences. For some participants, associations among variables changed in the span of some weeks, whereas they stayed stable over months for others. Our results further indicated nonstationarity in all participants.

**Conclusions:**

Idiographic symptom networks can provide insights into the temporal course of mental disorders and open new avenues of research for the study of the development and stability of psychopathological processes.

## Introduction

### Background

Different lines of research have established the heterogeneous nature of the etiology, clinical presentation, and treatment outcomes of depression [1-4], thus demonstrating a need for new ways to conceptualize and investigate the disorder. This is indicative of a broader issue across specific psychiatric diagnoses. The widespread evidence of substantial heterogeneity within diagnostic labels has increased the awareness of the need for more individualized research on mental disorders [5]. Although clinical psychology has a long tradition of interest in the individual, most studies in clinical psychology rely on nomothetic, cross-sectional data [5]. However, several theoretical arguments [6-8] and empirical studies [9] have shown that findings generated on a between-person basis are often not applicable to within-person processes, calling into question the extent to which cross-sectional studies are relevant for the understanding of individual clinical cases. Recently, the emergence of new theoretical approaches [10], statistical methods [11], and options for the collection of longitudinal data [12] have led to a surge in empirical studies of within-person, idiographic processes in clinical psychology [5].

Idiographic modeling of psychopathology has several possible advantages compared to group-level models. Owing to its potential to provide insights into temporal processes, an idiographic approach using longitudinal data could inform clinicians and researchers about the dynamics of psychological processes *of an individual* in a specific context [13], which is closely linked to clinical practice [14]. In combination with a focus on experiences in everyday life, idiographic models could lead to an improved understanding of mechanisms that influence the development and trajectory of mental disorders. During treatment, idiographic models could potentially be used to provide data-informed feedback to patients and therapists [13], develop personalized psychotherapy interventions [15], or design mobile interventions that are tailored to the individual [16].

To study such within-person processes, researchers use experience sampling methods to collect many observations per individual over time, also known as intensive longitudinal data [17]. Then, various forms of time series models can be used to investigate the relationships among multiple variables across time. Results of these models are often depicted as networks of variables that interact with one another. Then, these can be interpreted in accordance with the network approach of psychopathology that conceptualizes disorders as causal systems of mutually interacting symptoms [11,18]. Networks based on cross-sectional data have become very popular in the past few years, particularly in research on depression [19]. However, the so-called dynamic networks based on longitudinal data are especially promising for the network approach, as they both potentially allow insights into how disorders emerge from the interplay among individual symptoms over time and because they can reveal individual differences in symptom associations. Idiographic network models have, for example, been used to explore individual symptom patterns in different psychiatric disorders [20,21], including depression [22], to investigate psychotherapy processes [23] or to identify individualized treatment targets in eating disorders [24].

However, due to implicit assumptions of commonly used statistical methods, these patterns are typically modeled as static over time [25]. This approach restricts the investigation of change processes that can be of central interest to researchers and clinicians [14,26]. Therefore, most previous studies have not been able to investigate whether and how individual symptom networks change over time (for an early exception, refer to the study by Wichers and Groot [27]). For example, psychotherapists who are using daily diary data of their patients might be interested to examine whether the association between stress at work and subsequent depressive mood changes during therapy, as their patients might be incrementally able to handle stress better. This development would be difficult to account for when using typical models. In addition to this issue, experience sampling studies are often interested in variables that may change very fast, such as perceived stress or mood, and thus commonly follow individuals for a short time, often 1 or 2 weeks [28]. This study duration may be inappropriate for constructs such as depressive disorders, as it might miss slow changes developing over a longer time span, therefore incorrectly assuming that associations among symptoms are stable over time. We aimed to investigate the possibilities of circumventing these limitations by using recently developed methods for the estimation of time-varying models for psychological applications [25,29]. So far, this approach has not been used with time series data of multiple clinical cases.

### Objective

In this study, we applied time-varying network modeling to daily self-report data of patients diagnosed with recurrent depressive disorder to explore the idiographic course of depression over several months and to gain insight into the stability or instability of individual symptom networks of depression. In addition to 2 daily depressive core symptoms, namely anhedonia and feeling down, we included daily summaries of sleep duration, rumination, and the quality and quantity of social interactions as all these aspects have been hypothesized to interact in depression. We chose these items to gain multifaceted insight into the course of depression while limiting ourselves to a few variables for the demonstration and application of the chosen modeling technique. An in-depth theoretical background discussion regarding the selection of variables is provided in Multimedia Appendix 1 [25,29-58].

To justify the necessity of time-varying modeling, which is a more complex and data-intensive approach, we first tested whether the data-generating process of each individual in the time frame of our study was time varying by using a recently proposed hypothesis test [25]. Given the nonstationarity of participants’ time series, there were 2 main exploratory goals for this study. First, we aimed to construct individual networks for every patient to model the temporal associations among all variables on a day-to-day basis. Second, we wanted to explore the temporal dynamics of the individual course of depression by investigating changes in the network structure over time. To evaluate the initial indicators of trustworthiness of these models for use in the assessment of individual patients, we further aimed to assess the stability of estimates, prediction errors, and variance explained. The results of this study provide new insights into the time-varying nature of depression and highlight the usefulness and limitations of new statistical approaches to capture these temporal dynamics.

## Methods

### Transparency and Openness

This was, in part, a secondary analysis of the data previously analyzed by Lorenz et al [30] on the idiographic association between sleep and depression. Our analyses were preregistered after data collection and before secondary data analysis using the template for preregistration of experience sampling studies [59]. The preregistration and all code for the analyses can be accessed through Open Science Framework [60]. All deviations from the preregistered protocol are explained in detail in Multimedia Appendix 1.

### Ethical Considerations

The study was approved by the ethics committee of the University of Leipzig (258/17-ek). Participants who completed the data collection process were reimbursed €250 (approximately US $280) for their efforts for each study phase, implying a maximum individual financial compensation of €750 (approximately US $840). They could also keep the mobile phone that was provided to them for the study.

### Procedure

Data used in this study were collected as part of the research project, Sensor-Based System for Therapy Support and Management of Depression (STEADY). The overarching aim of the project was the creation of a sensor-based system for individuals with depression, integrating data from smartphones and wearable and stationary sensors to monitor the course of their disorder using self-assessments and physiological and behavioral markers. The data for this study were collected during a feasibility study of the STEADY system. The STEADY smartphone app was installed on a mobile phone that was provided to the participants for the completion of self-report protocols.

Data collection for the feasibility study was split into 3 consecutive study phases between 2017 and 2019, and they did not differ in their self-report protocols. An overview of all self-report questionnaires administered before, during, and after the study phases is provided in the preregistration. Participants were also given wrist-worn fitness trackers and stationary sleep sensors to collect passive sensing data. These data were not included in the analyses due to their questionable data quality. Within the smartphone app, participants were able to fill morning and evening logs. They were asked to complete them directly after waking up and shortly before going to bed, respectively. Morning logs were available from 3 AM to 3 PM, whereas evening logs were available from 3 PM to 3 AM.

Several precautionary measures were undertaken to assist in using the app and to prevent the occurrence of missing data, such as monthly visits to the study center and phone calls.

### Participants

Participants were recruited in cooperation with the Department of Psychiatry and Psychotherapy (University of Leipzig Medical Center, Germany). Potential participants were informed about the study by their treating physicians. If they indicated interest, they were contacted by a staff member of the study center, who conducted the formal examination of inclusion and exclusion criteria. If the individual was found to be eligible, written consent for participation was obtained from them. During the initial diagnostic screening, invitees were inquired about sociodemographic information and their medical history in a semistructured interview. The Structured Clinical Interview for Diagnostic and Statistical Manual of Mental Disorders–4 [61] (in its German translation [62]) was used to assess psychiatric diagnoses. The Inventory of Depressive Symptomatology, Clinician Rated (IDS-C) [63] (in its German translation [31]) was used by trained raters to assess current depressive symptom severity.

Inclusion criteria were the following: having a diagnosis of a recurrent depressive disorder; having a current depressive symptom level of at least 14 points on the IDS-C; currently being treated professionally for depression; being aged at least 18 years; and living near the research center of the German Depression Foundation in Leipzig, Germany, to accommodate regular in-person appointments. Individuals were excluded if they had severe somatic disorders; displayed acute suicidal behavior; were pregnant or in the lactation period; had electronic implants; or experienced the following psychiatric comorbidities: borderline personality disorder, schizophrenia, alcohol or drug addiction, or schizotypal and delusional disorders. For this study, we prespecified that participants should have at least 130 days of data and <30% missingness for any variable. Of the 25 total participants, we included 20 (80%) individuals in our analyses. A detailed description of the sample is provided in the following sections.

### Measures

#### Pre-Post Assessment of Depressive Symptom Levels

The IDS-C [63] was used to describe depressive symptom levels before and after the data collection period. It was assessed before and after daily data collection and during some of the monthly visits, but data from only 1 questionnaire each, before the start and after the end of an individual time series, was used in this paper. The IDS-C consists of 30 items that inquire about a range of depressive symptoms, of which 28 items (scored from 0-3) were included in this paper. Items of the IDS-C are weighted equally and combined into a sum score ranging from 0 to 84, where a cutoff point of 13 was originally proposed to identify individuals with symptoms [63]. The IDS-C has been evaluated psychometrically in different populations, including individuals with depression, in both its original version and its German translation [32].

#### Daily Diary Measures

A variety of self-report questions was used in the morning and evening protocols. As preregistered, we chose a subset of all self-report variables for the following reasons: theoretical relevance for depression, assessment on a continuous scale, sampling frequency, frequency of missingness, and a general preference for sparsity for our estimation method. In total, 6 daily items were used in this study: loss of interest or joylessness, feeling down or depressed or hopeless, rumination, quantity of social contacts, quality of social contacts, and sleep duration (refer to Multimedia Appendix 1 for the wording).

The first 2 items represent depressive symptoms resembling the items of the Patient Health Questionnaire–2 [64] and are listed as core symptoms in the *Diagnostic and Statistical Manual of Mental Disorders, Fifth Edition* [65]. For this study, these items were reframed to inquire about a single day. They were described as *anhedonia* for the first item and *feeling down* for the second item. The quantity and quality of social contacts were also assessed using a visual analog scale, where participants could indicate how many contacts they had and how they felt about these social contacts. All these items were assessed in the evening logs. Total sleep time was assessed in the morning logs. Participants were asked about when they went to bed and when they got up in the morning. The time spent in bed was calculated by the app, and participants were then asked how much of this time they spent sleeping. The items used in this study have not been psychometrically evaluated but were specifically created for the STEADY app. In addition to daily questions, some of the participants provided qualitative information regarding significant life events during monthly visits to the study center.

### Data Preparation and Statistical Analysis

#### Overview

The software environment R (version 4.1.1; R Foundation for Statistical Computing) [66] was used for all analyses in the study. Version control information and the R code for all analyses can be accessed through the Open Science Framework repository connected to this project [60]. All variables in the data set were treated as continuous variables.

#### Missing Data

Owing to the long data collection period, several participants had many blocks of consecutive data separated by an extended period of missingness. To obtain the single longest time series for each participant, we searched for the longest phase without item-wise missing data for >7 consecutive days and discarded the remaining data. We chose a maximum window of consecutive missingness as we did not want to impute several consecutive missing data points. A detailed workflow for handling missing data is available in the preregistration. We performed item-wise missing value imputation using the Kalman filter, which has been shown to perform well in a previous simulation study of idiographic network analysis [33]. We used the Kalman filter from the R package, *imputeTS* [67], in its default setting.

#### Statistical Analysis

##### Time-Varying Vector Autoregression

Vector autoregressive (VAR) models are time series models that can be used to investigate the relationships among multiple variables at a given lag size. For example, in a VAR model of lag 1, the value of a variable at a given time point is regressed on the value of itself (known as autoregressive effect) and all other variables (known as cross-lagged effects) at the previous time point. As mentioned in the *Introduction* section, these models assume stationarity, meaning that the parameters of the model are assumed to be constant over time, which may not be appropriate for many research questions. Different techniques have been applied to explicitly account for time-varying parameters in psychological time series in the past, mostly focusing on univariate or bivariate associations [23,68-70]. Recently, a new approach for estimating time-varying VAR models based on kernel smoothing has been developed and tested in a simulation study [25,29]. Using VAR models with kernel smoothing allows the estimation of parameters that change over time and choosing between models with different degrees of flexibility to vary over time.

We used time-varying VAR models as implemented in the R package, *mgm* [29], to estimate idiographic models with a default lag size of 1 for reasons of parsimony. Further details about the method and our implementation of the model are available in Multimedia Appendix 1 and in the papers by Haslbeck et al [25] and Haslbeck and Waldorp [29]. To obtain the time-varying parameters, local VAR models are estimated at several equidistant estimation points and then combined. In these local models, observations closer to a specific estimation point are weighted more strongly than observations farther away. The kernel weighting used to achieve this is characterized by its bandwidth, which determines the number and weights of observations included in the estimation. We have described and visualized the resulting models as dynamic networks. These comprise *nodes* (representing variables) and *edges* (representing the temporal associations among variables). The networks were visualized using the R package, *qgraph* [71].

##### Bandwidth Selection

Bandwidth selection represents a bias-variance trade-off [72], where smaller bandwidths lead to highly local estimates and faster changes. Large bandwidths >1 lead to an estimation that is increasingly similar to the results of estimating a stationary model [25]. To select an appropriate individual bandwidth, several candidate bandwidths were compared using a time-stratified, 5-fold, cross-validation scheme. Then, we selected the bandwidth that minimized the root mean squared error (RMSE) across the test sets. Details about the bandwidth selection scheme and an exploratory analysis of the robustness of selection are available in Multimedia Appendix 1. A visual illustration of the difference between bandwidths of different sizes is provided in Multimedia Appendix 1.

##### Model Estimation

Using the selected bandwidth, parameter estimates for every estimation point were then obtained via regularized regression using the *least absolute shrinkage and selection operator* (lasso) [73]. The lasso is a regularization technique that shrinks parameter estimates while possibly setting some of them to 0. The choice of regularization parameters is explained in Multimedia Appendix 1. As the lasso is sensitive to different variances, we z-transformed all the variables before all the analyses. Following the simulation study by Haslbeck et al [25], our final model was estimated using 20 equally spaced estimation points. We distinguished the term estimation point from the term time point, which refers to a single daily observation.

##### Stability and Predictability

To gain insight into the stability of the parameter estimates, we used a block bootstrap scheme to obtain bootstrapped sampling distributions. We further computed the proportion of explained variance (*R*^2^) and RMSE as prediction errors for each variable at each estimation point. To accomplish this, we used the weighted method of forming a prediction error as implemented in the *mgm* package. The *R*^2^ values for all participants at all estimation points are available in Multimedia Appendix 1.

##### Stationarity Hypothesis Test

To test whether the data-generating process of the time series of an individual was stationary, we implemented a significance test as proposed by Haslbeck et al [25] to test the null hypothesis that the process was stationary. Details about the test are available in Multimedia Appendix 1.

## Results

### Sample Characteristics

The 20 included participants (n=13, 65% women) had a mean age of 44.4 (SD 11.6; range 26-67) years during screening. Data were available for 274 (SD 82.4; range 154-539) days (ie, time points) on average. In the selected time series of participants, 5.53% of the data were missing*.* Further information about the missing data structure is provided in Multimedia Appendix 1. Of the 20 participants, 18 (90%) were German citizens, 1 (5%) had dual citizenship, and 1 (5%) had a different nationality*.* Of the 20 participants, 11 (55%) had completed the general higher education entrance qualification. On average, IDS-C values decreased from 26 before the start of the time series to 22.9 afterward. Of the 20 participants, 18 (90%) took antidepressant medication and 18 (90%) currently or previously underwent psychotherapeutic treatment*.* More information about every participant is provided in Table 1.

**Table 1:** Sample characteristics.

ID^a^	Sex	Age range (y)^b^	Time series length, n	Missingness (%)^c^	IDS-C^d^ score before EMA^e^ data collection	IDS-C score after EMA data collection	Bandwidth
1	Female	40-45	311	12.6	24	10	0.45
2	Female	45-50	539	5.0	24	11	0.009
3	Male	50-55	308	3.7	38	30	0.12
4	Female	30-35	204	14.5	35	18	0.12
5	Female	40-45	205	3.8	19	1	0.12
6	Female	60-65	301	2.3	34	40	0.12
7	Male	40-45	309	6.9	7	2	0.23
8	Female	40-45	283	1.8	35	29	0.01
9	Female	35-40	189	2.9	19	38	0.01
10	Female	30-35	316	4.2	11	12	0.01
11	Male	40-45	204	1.0	19	9	0.12
13	Male	50-55	303	1.8	41	47	0.12
15	Male	50-55	263	8.1	28	57	0.01
16	Female	30-35	301	3.7	23	6	0.01
17	Female	30-35	154	2.9	27	21	0.12
18	Female	50-55	198	19.6	17	21	0.23
19	Female	25-30	303	2.7	20	23	0.01
20	Female	65-70	301	4.7	25	23	0.12
21	Male	25-30	194	7.2	27	25	1.00
22	Male	60-65	302	1.4	47	35	0.12

^a^Only individuals who were included in the analyses for this study.

^b^We provided age ranges to prevent the identifiability of participants.

^c^Missingness (%) reflects the individual percentage of item-wise missing data averaged over all variables.

^d^IDS-C: Inventory of Depressive Symptomatology, Clinician Rated.

^e^EMA: ecological momentary assessment.

### Stationarity Tests

The test for stationarity led to the rejection of the null hypothesis for all participants (20/20, 100%), meaning that we rejected the hypothesis that the data-generating process of an individual time series was stationary. The results for all participants are available in Multimedia Appendix 1. These results provided us a first indication of the necessity of using a time-varying approach for our data.

### Case Studies

#### Overview

In the following sections, we have presented the individual results of 2 participants (participants 6 and 11). Participant 6 was chosen because she provided qualitative information about crucial life events during her time series, whereas the network structure of participant 11 changed alongside a reported improved depressive symptomatology over time. We have presented individual networks at estimation points 2, 10, and 19 to showcase models at the beginning, in the middle, and at the end of the time series. We chose to present the 3 edges with the highest intraindividual variability. We have included a case study of participant 2 in Multimedia Appendix 1 to showcase the potential shortcomings of the method that we used.

#### Participant 6

**Figure 1:**
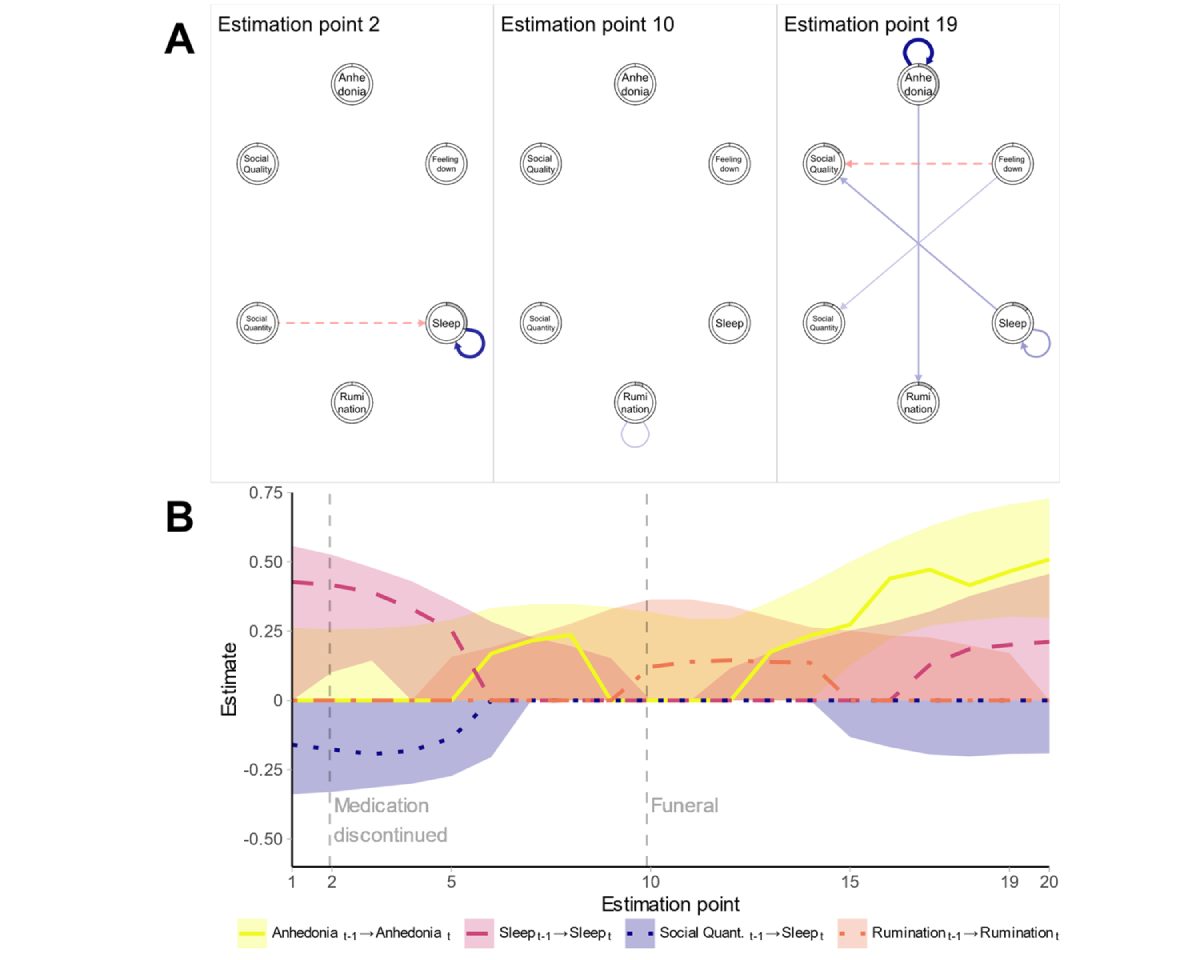
Results for participant 6: (A) networks at estimation points 2, 10, and 19; (B) time-varying parameters.

Participant 6 was a German woman in her 60s, in partial retirement, with a comorbid anxiety disorder. She started multiple antidepressant medications in the year before the study began. She also had previously undergone several psychotherapeutic treatments, and she underwent psychotherapy at the beginning of data collection. A bandwidth of 0.12 was selected for her time series with a final length of 301 days. Her results are visualized in Figure 1.

The networks at estimation points 2, 10, and 19 (corresponding to days 17, 143, and 285) are displayed in Figure 1A. Width and saturation of edges in the networks were scaled with respect to an arbitrary maximum of 0.5 for all participants, as approximately 80% of all absolute non-0 edge weights were below this threshold. Time-varying parameters are shown in Figure 1B. Estimates at the end of the time series need to be interpreted with caution as fewer data are available for that period. Effects are sorted based on the extent of variability over time in all plots—the effect with the highest variability is plotted in yellow, the effect with the second-highest variability is plotted in violet, and the effect with the third-highest variability is plotted in blue. The autoregressive effect of rumination in orange was added irrespective of its extent of variability over time.

Immediately before the start of the time series, the participant experienced panic attacks, and the dosage of her medication was increased. A few days before the second estimation point (approximately 32 days after the beginning of the time series), she started to phase out her medication, in other words, she continuously reduced the dosage. Both the negative cross-lagged effect from the quantity of social contacts on sleep and the positive autoregressive effect of sleep subsequently disappeared. As self-report sleep involves a different time interval (night) than the remaining measures that summarize the day, the cross-lagged effects including sleep are only interpretable in an asymmetric fashion. In this example, social contacts had an effect on sleep during the subsequent night. Shortly before the 10th estimation point, the participant reported that she visited a funeral and worried about a friend of hers. After that, an autoregressive effect of rumination was detected, which can be interpreted as a prolonged tendency to stay in a ruminative thought process. No more qualitative information about life events was available after this time point. As with many other participants, the bootstrapping results, which we did not include in this paper to keep the plot from being very visually cluttered, indicated a substantial uncertainty of point estimates. This implies that the specific numerical results of an association should be interpreted with caution, as they are likely unstable.

At the end of the time series, the network of participant 6 was relatively strongly connected, with a strong autoregressive effect of anhedonia. Feeling down was positively associated with the quantity of next-day social contacts but negatively associated with their perceived quality. Her IDS-C sum score increased from 34 to 40 from the beginning to the end of the time series, indicating a worsening of her symptomatology. The average *R*^2^ value averaged over all items and estimation points was 0.113, reflecting many estimation points with almost no associations, which we observed for many participants with smaller bandwidths. *R²* was lowest in the middle of the time series and highest at the end. While the networks of these participants were estimated to be empty at many estimation points, the remaining networks changed very fast over time such that most estimated edges were present only for a short time. In some cases, edges even became inverted within a few estimation points, such that the association between 2 variables was positive at one estimation point and negative at another.

#### Participant 11

**Figure 2:**
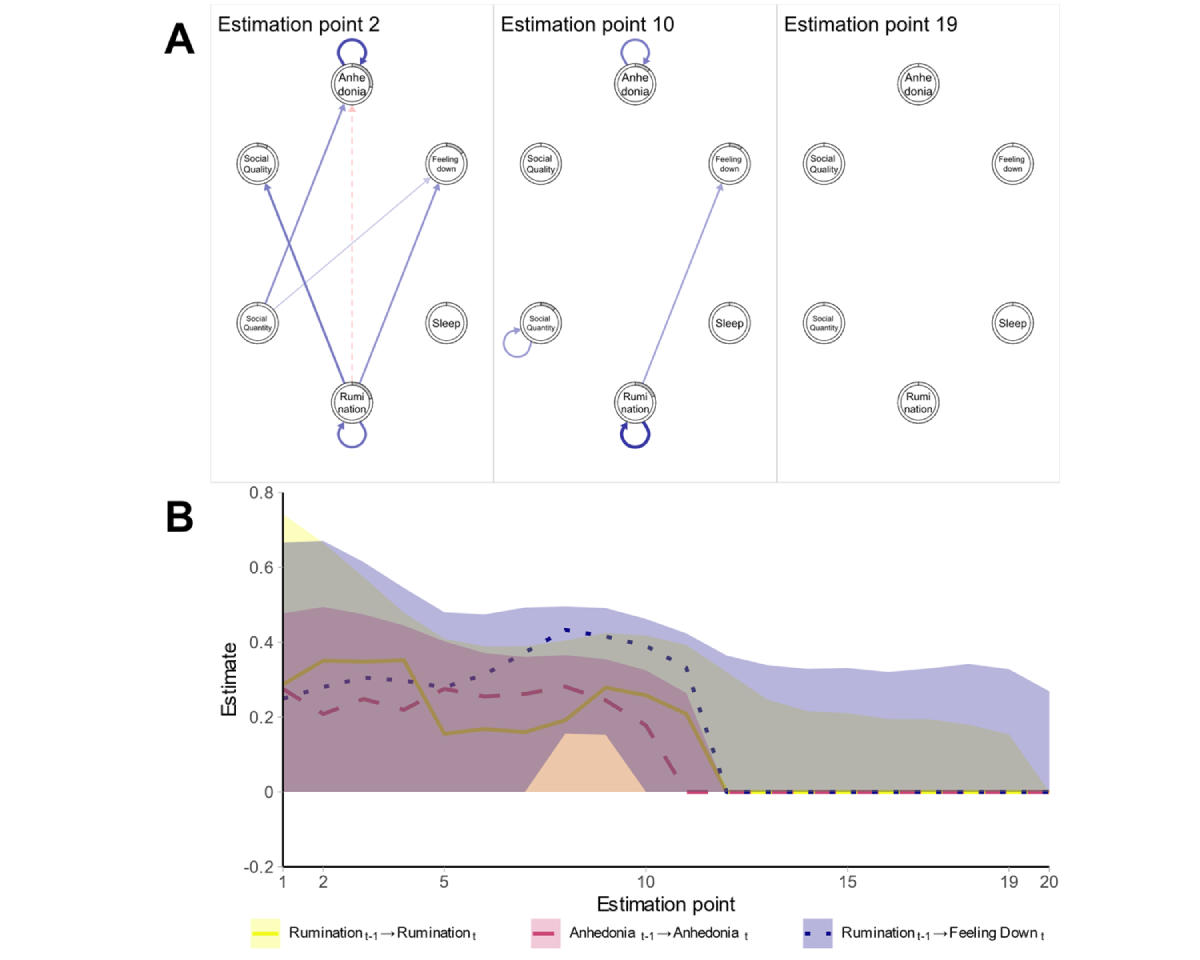
Results for participant 11: (A) networks at estimation points 2, 10, and 19; (B) time-varying parameters.

Participant 11 was a self-employed German man in his 40s, who was prescribed an antidepressant medication to treat his depression. He did not previously access any psychotherapeutic services. His time series spanned 204 days, and a bandwidth of 0.12 was selected for his data. Again, the estimated networks and visualization of 3 parameters over time are presented in Figure 2.

The networks at estimation points 2, 10, and 19 (corresponding to days 12, 97, and 193) are displayed in Figure 2A. Time-varying parameters are shown in Figure 2B. The y-axis in this plot is different from that in Figure 1 to accommodate the large width of the bootstrapped sampling distribution.

At the start of the time series, the network of participant 11 was strongly connected. The effect of rumination on depressive symptoms diverged in the beginning, with a negative effect on next-day anhedonia and a positive effect on feeling down. Both anhedonia and rumination showed strong autoregressive effects at the beginning of the time series. This indicates a resistance to change for these variables, meaning that if a larger deviation from their expected value occurs, it takes longer for these variables to return to their “normal” values. Over time, most of these effects became weaker and ultimately disappeared, whereas only autoregressive effects and a positive association of rumination with next-day feeling down remained. At some estimation points, a weak, positive autoregressive effect of both social variables and a positive effect of quality on the quantity of next-day social contacts emerged (not depicted in this paper). In the end, the network of participant 11 became empty, which was also observed in other participants (such as participants 1, 5, and 18). In addition, depressive symptomatology, as measured using the IDS-C, decreased from 19 to 9, which is an improvement to what typically would be judged as a subclinical symptom level. The average *R*^2^ value over time points and variables was 0.228. It decreased toward the end, reflecting the empty networks estimated at the end of the time series. All the bootstrapped sampling distributions around the point estimates pointed toward a strong instability of the point estimates, showing that the interpretation of point estimates warrants caution.

### Model Quality

In addition to focusing on the individual associations among variables and their change over time, we investigated model fit indices and the raw distribution of variables to check the overall quality of our models and possible violations of assumptions. The RMSE and *R*^2^ values were computed for every variable of each participant at all time points. Averaged over all participants, the mean RMSE was 0.865 (SD 0.111), and the mean *R*^2^ value was 0.235 (SD 0.181). Participant 9 showed the best fit, with an RMSE of 0.714 and an *R*^2^ value of 0.486, whereas participant 3 showed the worst fit, with an RMSE of 0.972 and an *R*^2^ value of 0.054. Overall, this indicates large differences in model fit among participants, with some models showing very poor fit, whereas others had a relatively good fit to the data. There are many potential reasons for these differences, such as overfitting or underfitting of the models or characteristics of the data that violate the assumptions of the model such as strong nonnormality or abrupt changes.

For 30% (6/20) of the participants, we observed strong floor or ceiling effects in at least 1 item, commonly in either core depressive or social contact items, with the relative frequencies of maximum or minimum scale values exceeding 50%. As these highly skewed data violated the model assumptions, we chose not to interpret the resulting networks of these participants further. However, participant 6 also showed a ceiling effect for the first depression item, as she answered “all the time” for approximately 54.8% (165/301) of the days. Nevertheless, we chose to present her results, as none of the other items were affected, and she provided more relevant qualitative information than any other participant.

## Discussion

### Principal Findings

The goal of this study was to model the idiographic temporal dynamics of depressive symptoms and other variables associated with depression using daily diary data to gain insight into the temporal dynamics of the disorder. Therefore, we used a recently developed modeling technique that allows for the estimation of time-varying parameters. Both the results of our hypothesis tests and the bandwidth selection procedure provided evidence of substantial changes over time for most participants and thus supported the use of a technique that accounts for these dynamics. We described our results as networks of mutually influencing variables and highlighted the changes in the connections among them over time for exemplar participants. Our results showed extensive variation over time for some participants and marked variability among the networks of different individuals, whereas the bootstrap results suggested the general instability of point estimates.

Individual networks showed temporal associations that might be useful for clinical interpretation and use in self-monitoring contexts. A positive autoregressive effect of rumination, which was present for some estimation points for participants 6 and 11, is sometimes termed as ruminative inertia [74]. Becoming stuck in rumination might be a relevant cognitive mechanism that explains the negative influences of rumination on depression [74]. The contrasting effect of rumination on both depressive symptoms for participant 11 at some estimation points highlights the notion that certain aspects of rumination could also be adaptive for this individual at some times and could therefore differentially impact depressive symptoms. Regarding participant 6, observing a positive effect of feeling down on next-day social quantity at the same time as a negative effect on next-day social quality at the end of her time series could be interpreted as seeking more social contacts after days when she felt depressed, possibly as a remedy or coping strategy, but still being less able to enjoy them or shape them positively.

These person-specific relationships could then be translated into a treatment context by discussing them with the patient or by using them as hypothesis-generating models for potential intervention targets [75]. In addition to specific temporal associations, node-wise summary measures such as node centrality or predictability have been discussed as potential indicators of the relevance of a certain symptom and, subsequently, as potential guides for intervention targets [24,26]. Time-varying networks provide a potential advantage over time-invariant networks in that they could be leveraged for just-in-time adaptive interventions [76]. For example, if the increase of a certain symptom or behavior strongly predicts increased depressive symptomatology, such information could be used to generate personalized interventions. We have discussed the statistical issues and potential solutions related to this topic in the following sections.

As evident in the provided examples, the results of bandwidth selection and in our hypothesis tests, we observed a strong variation in parameter estimates over time, which highlights the substantial variability of symptom interrelations within a person over the course of their depression. This is consistent with the results obtained by Howe et al [70], who found strong intraindividual variation in the networks among different mood states of participants over time. As they discussed, this finding indicates that when associations among symptoms are not time invariant, interventions based on an analysis of symptom-level associations that do not take this variation over time into account might be suboptimal. This could be especially relevant for the development of personalized, just-in-time adaptive interventions, which aim to provide personalized interventions at the right time. For example, if the quantity of social contacts showed a negative predictive association with depressive mood at some point but a positive one at another, an intervention to increase social activity might not always be beneficial. Thus, our results again reinforce recent calls for the use of network analysis methods that are equipped to detect variation over time [26] to better understand the dynamic nature of mental disorders. Additional qualitative information, as we presented for participant 6, could provide important information to interpret the changes in parameters over time and thus increase the clinical utility of the method.

The general decrease in the number and strength of next-day associations alongside an improvement in depressive symptomatology, which was evident for both participant 11 and other participants not shown in this paper, lends itself to an interpretation from a dynamical systems perspective about depression. The idea that individuals with more strongly connected depressive symptoms are more susceptible to ending up in a depressive state (put forth by Cramer et al [77]) has been investigated in various populations and contexts [78]. The time-varying approach used in this study could provide an interesting, new perspective on these issues [25]. The exemplars presented in this paper are not meant to provide any substantial evidence on the general question of the role of network connectivity in mental disorders. Decreased connectivity was, for example, also observed for participant 18, whose symptoms worsened slightly.

Although we can draw interesting insights from individual networks and their change over time, results for participants with a small bandwidth (refer to Multimedia Appendix 1 for a detailed case study) stand out because parameters changed fast and many networks were empty. While it is possible that the symptomatology of these individuals changes quickly and that no days, weeks, or months are alike, there are various other, at least equally plausible reasons for these results. These include the possibility of fundamental issues with assessment, such as the inappropriateness of our measures or inaccurate responses [79]. Irrespective of its root cause, the nature of these results can hamper the usefulness of this method. We have discussed the possible technical solutions to this issue in Multimedia Appendix 1, but general issues with power and interpretability remain notwithstanding.

These considerations point to a more general question: in which contexts can time-varying models be useful? Simulation results have shown that time-varying models can outperform stationary ones even at a low number of observations of approximately 50, under certain conditions [25]. Thus, in principle, these models are both applicable to research, where one may be interested in finding specific time-varying phenomena and clinical contexts, where change over time may be interesting information as feedback for clinicians or patients. To make them useful in the latter case, choosing an appropriate context where gradual change is to be expected is important. In the case of changes due to major life events reported by participants, time-varying models that can accommodate abrupt changes might be an appropriate choice [80]. As time-varying techniques need a large amount of data, the proper selection of a limited number of variables is crucial. The need for a large number of observations per individual can be easier to achieve with passively collected data, such as from fitness trackers or smartphone data. When a large amount of data are available and there is a lack of theoretical knowledge about the form of relationships among variables, time-varying models might prove to be especially useful. In summary, when sufficient data can be collected and some gradual change among variables is to be expected and is of interest, time-varying models can shine.

However, although idiographic network models and time-varying subtypes are promising approaches, their clinical utility has not yet been established [81]. In general, idiographic network models have only been applied to clinical practice in small pilot studies [82]. Models that use purely data-driven approaches based on data from a single individual have several limitations, as they can ignore clinical judgment and can be difficult to estimate and interpret properly [34,83]. These issues can be counteracted by integrating clinical knowledge [83] or information from other individuals into individual networks [33,84]. These potential drawbacks of a purely idiographic approach may seem contradictory to the information in the Introduction section, where the nomothetic-idiographic divide and the advantages of the latter were emphasized. Instead of adopting an either-or perspective, highly person-specific approaches with intensive data collection such as those presented in this paper can still be crucial to provide individual feedback and to detect phenomena that would be obscured with less granular methods, thus serving as building blocks for nomothetic studies that aim to generalize these results.

### Strengths and Limitations

Our study had several considerable strengths. We used an innovative modeling technique to explicitly model time-varying parameters in symptom networks. The combination of long individual time series with relatively few missing data and the low number of variables, which is desirable for the performance of the presented method, was conducive to the quality of estimation. Furthermore, our detailed preregistration and open code provide transparency for other researchers.

However, the psychological processes that we are interested in are complex and can occur on a variety of timescales, and our assessments of those processes are affected by measurement error [85,86]. Therefore, our models should be interpreted with several caveats in mind. In general, estimated effects are strongly dependent on which variables are present or absent in the network [87], implying that the inclusion of more depressive symptoms would possibly change our results. Furthermore, the results of parameter estimation crucially depend on the chosen sampling frequency [88] and the subsequent lag choice [17]. Regarding measurement, the use of single items to assess psychological constructs has psychometric disadvantages [14] and may obfuscate the inherent heterogeneity of what likely should not be considered as homogenous constructs (refer to the paper by Bernstein et al [35] for the example of rumination). Moreover, the specific items used in this study were not previously assessed for validity or reliability.

A large number of empty or very sparse networks point to two further limitations of our method. In general, idiographic network models often experience power problems [33], which are further exacerbated in the power-hungry estimation of time-varying networks. Relatedly, regularization techniques decrease sensitivity and prohibit the construction of conventional CIs (refer to the paper by Williams et al [89] and Williams [90] for a discussion regarding lasso in network estimation).

### Future Directions

There are multiple avenues for further studies. First, applied researchers can use time-varying VAR models in scenarios where substantial change can be expected and is of core interest, and repeated intensive assessment is feasible to better understand the temporal development of mental disorders. Second, building on its potential clinical use, further methodological research into the estimation method used in this study could provide more information on best practices regarding modeling options. Third, we observed the potential utility of qualitative information as context for time series data. Thus, further studies could investigate best practices regarding the collection and integration of qualitative information into intensive, longitudinal designs and analyses.

### Conclusions

Attempts to develop personalized models of psychopathology have become increasingly refined in recent years. While there have been large advances regarding the modeling of interindividual variation, studies of variation within individuals with high temporal solution have lagged. We have made a step forward in this direction by explicitly modeling individual changes in the associations among depressive symptoms over time. Pronounced within-person variation in our results highlights the importance of investigating the temporal dynamics of symptoms and their interplay over time.

## Appendix

Multimedia Appendix 1 Supplementary materials.

## Abbreviations

IDS-C: Inventory of Depressive Symptomatology, Clinician Rated
lasso: least absolute shrinkage and selection operator
RMSE: root mean squared error
STEADY: Sensor-Based System for Therapy Support and Management of Depression
VAR: vector autoregressive

## References

[1] Cai N, Choi KW, Fried EI (2020). Reviewing the genetics of heterogeneity in depression: operationalizations, manifestations and etiologies. Hum Mol Genet.

[2] Fried EI (2015). Problematic assumptions have slowed down depression research: why symptoms, not syndromes are the way forward. Front Psychol.

[3] Kessler RC, van Loo HM, Wardenaar KJ, Bossarte RM, Brenner LA, Ebert DD, de Jonge P, Nierenberg AA, Rosellini AJ, Sampson NA, Schoevers RA, Wilcox MA, Zaslavsky AM (2017). Using patient self-reports to study heterogeneity of treatment effects in major depressive disorder. Epidemiol Psychiatr Sci.

[4] Maj M, Stein DJ, Parker G, Zimmerman M, Fava GA, de Hert M, Demyttenaere K, McIntyre RS, Widiger T, Wittchen HU (2020). The clinical characterization of the adult patient with depression aimed at personalization of management. World Psychiatry.

[5] Wright AG, Woods WC (2020). Personalized models of psychopathology. Annu Rev Clin Psychol.

[6] Borsboom D, Mellenbergh GJ, van Heerden J (2003). The theoretical status of latent variables. Psychol Rev.

[7] Hamaker EL, Mehl MR, Conner TS (2012). Why researchers should think "within-person": a paradigmatic rationale. Handbook of Research Methods for Studying Daily Life.

[8] Molenaar PC (2004). A manifesto on psychology as idiographic science: bringing the person back into scientific psychology, this time forever. Meas Interdiscip Res Perspect.

[9] Fisher AJ, Medaglia JD, Jeronimus BF (2018). Lack of group-to-individual generalizability is a threat to human subjects research. Proc Natl Acad Sci U S A.

[10] Borsboom D (2017). A network theory of mental disorders. World Psychiatry.

[11] Bringmann LF, Vissers N, Wichers M, Geschwind N, Kuppens P, Peeters F, Borsboom D, Tuerlinckx F (2013). A network approach to psychopathology: new insights into clinical longitudinal data. PLoS One.

[12] Shiffman S, Stone AA, Hufford MR (2008). Ecological momentary assessment. Annu Rev Clin Psychol.

[13] Hayes SC, Hofmann SG, Stanton CE, Carpenter JK, Sanford BT, Curtiss JE, Ciarrochi J (2019). The role of the individual in the coming era of process-based therapy. Behav Res Ther.

[14] Piccirillo ML, Beck ED, Rodebaugh TL (2019). A clinician’s primer for idiographic research: considerations and recommendations. Behav Ther.

[15] Rubel JA, Fisher AJ, Husen K, Lutz W (2018). Translating person-specific network models into personalized treatments: development and demonstration of the dynamic assessment treatment algorithm for individual networks (DATA-IN). Psychother Psychosom.

[16] Wang L, Miller LC (2020). Just-in-the-moment adaptive interventions (JITAI): a meta-analytical review. Health Commun.

[17] Hamaker EL, Wichers M (2017). No time like the present: discovering the hidden dynamics in intensive longitudinal data. Curr Dir Psychol Sci.

[18] Borsboom D, Cramer AO (2013). Network analysis: an integrative approach to the structure of psychopathology. Annu Rev Clin Psychol.

[19] Wichers M, Riese H, Hodges TM, Snippe E, Bos FM (2021). A narrative review of network studies in depression: what different methodological approaches tell us about depression. Front Psychiatry.

[20] David SJ, Marshall AJ, Evanovich EK, Mumma GH (2018). Intraindividual dynamic network analysis - implications for clinical assessment. J Psychopathol Behav Assess.

[21] Fisher AJ, Reeves JW, Lawyer G, Medaglia JD, Rubel JA (2017). Exploring the idiographic dynamics of mood and anxiety via network analysis. J Abnorm Psychol.

[22] Rodriguez M, Aalbers G, McNally RJ (2021). Idiographic network models of social media use and depression symptoms. Cogn Ther Res.

[23] Kaiser T, Laireiter AR (2018). Process-symptom-bridges in psychotherapy: an idiographic network approach. J Pers Oriented Res.

[24] Levinson CA, Hunt RA, Keshishian AC, Brown ML, Vanzhula I, Christian C, Brosof LC, Williams BM (2021). Using individual networks to identify treatment targets for eating disorder treatment: a proof-of-concept study and initial data. J Eat Disord.

[25] Haslbeck JM, Bringmann LF, Waldorp LJ (2021). A tutorial on estimating time-varying vector autoregressive models. Multivariate Behav Res.

[26] Bringmann LF, Albers C, Bockting C, Borsboom D, Ceulemans E, Cramer A, Epskamp S, Eronen MI, Hamaker E, Kuppens P, Lutz W, McNally RJ, Molenaar P, Tio P, Voelkle MC, Wichers M (2022). Psychopathological networks: theory, methods and practice. Behav Res Ther.

[27] Wichers M, Groot PC, Psychosystems, ESM Group, EWS Group (2016). Critical slowing down as a personalized early warning signal for depression. Psychother Psychosom.

[28] Wrzus C, Neubauer A Ecological momentary assessment: a meta-analysis on designs, samples, and compliance across research fields. PsyArXiv.

[29] Haslbeck JM, Waldorp LJ (2020). mgm: estimating time-varying mixed graphical models in high-dimensional data. J Stat Softw.

[30] Lorenz N, Sander C, Ivanova G, Hegerl U (2020). Temporal associations of daily changes in sleep and depression core symptoms in patients suffering from major depressive disorder: idiographic time-series analysis. JMIR Ment Health.

[31] Drieling T, Schärer LO, Langosch JM (2007). The inventory of depressive symptomatology: German translation and psychometric validation. Int J Methods Psychiatr Res.

[32] Boden S (2018). Diagnostik von Depressivität: Validierung des Inventars depressiver Symptome (IDS). Eberhard Karl University of Tübingen.

[33] Mansueto AC, Wiers RW, van Weert JC, Schouten BC, Epskamp S (2023). Investigating the feasibility of idiographic network models. Psychol Methods.

[34] Klintwall L, Bellander M, Cervin M (2023). Perceived causal problem networks: reliability, central problems, and clinical utility for depression. Assessment.

[35] Bernstein EE, Heeren A, McNally RJ (2019). Reexamining trait rumination as a system of repetitive negative thoughts: a network analysis. J Behav Ther Exp Psychiatry.

[36] Nolen-Hoeksema S, Wisco BE, Lyubomirsky S (2008). Rethinking rumination. Perspect Psychol Sci.

[37] Olatunji BO, Naragon-Gainey K, Wolitzky-Taylor KB (2013). Specificity of rumination in anxiety and depression: a multimodal meta‐analysis. Clin Psychol Sci Pract.

[38] Watkins E (2015). Psychological treatment of depressive rumination. Curr Opin Psychol.

[39] Ebrahimi OV, Burger J, Hoffart A, Johnson SU (2021). Within- and across-day patterns of interplay between depressive symptoms and related psychopathological processes: a dynamic network approach during the COVID-19 pandemic. BMC Med.

[40] Lunansky G, van Borkulo CD, Haslbeck JM, van der Linden MA, Garay CJ, Etchevers MJ, Borsboom D (2021). The mental health ecosystem: extending symptom networks with risk and protective factors. Front Psychiatry.

[41] Hirschfeld RM, Montgomery SA, Keller MB, Kasper S, Schatzberg AF, Möller HJ, Healy D, Baldwin D, Humble M, Versiani M, Montenegro R, Bourgeois M (2000). Social functioning in depression: a review. J Clin Psychiatry.

[42] Gariépy G, Honkaniemi H, Quesnel-Vallée A (2016). Social support and protection from depression: systematic review of current findings in Western countries. Br J Psychiatry.

[43] Brown LH, Strauman T, Barrantes-Vidal N, Silvia PJ, Kwapil TR (2011). An experience-sampling study of depressive symptoms and their social context. J Nerv Ment Dis.

[44] Elmer T, Stadtfeld C (2020). Depressive symptoms are associated with social isolation in face-to-face interaction networks. Sci Rep.

[45] Chevance A, Ravaud P, Tomlinson A, Le Berre C, Teufer B, Touboul S, Fried EI, Gartlehner G, Cipriani A, Tran VT (2020). Identifying outcomes for depression that matter to patients, informal caregivers, and health-care professionals: qualitative content analysis of a large international online survey. Lancet Psychiatry.

[46] Elmer T, Geschwind N, Peeters F, Wichers M, Bringmann L (2020). Getting stuck in social isolation: solitude inertia and depressive symptoms. J Abnorm Psychol.

[47] Fried EI (2017). The 52 symptoms of major depression: lack of content overlap among seven common depression scales. J Affect Disord.

[48] Baglioni C, Nanovska S, Regen W, Spiegelhalder K, Feige B, Nissen C, Reynolds CF, Riemann D (2016). Sleep and mental disorders: a meta-analysis of polysomnographic research. Psychol Bull.

[49] Nutt D, Wilson S, Paterson L (2022). Sleep disorders as core symptoms of depression. Dialogues Clin Neurosci.

[50] Alvaro PK, Roberts RM, Harris JK (2013). A systematic review assessing bidirectionality between sleep disturbances, anxiety, and depression. Sleep.

[51] Cunningham JE, Shapiro CM (2018). Cognitive behavioural therapy for insomnia (CBT-I) to treat depression: a systematic review. J Psychosom Res.

[52] Pillai V, Steenburg LA, Ciesla JA, Roth T, Drake CL (2014). A seven day actigraphy-based study of rumination and sleep disturbance among young adults with depressive symptoms. J Psychosom Res.

[53] Manea L, Gilbody S, Hewitt C, North A, Plummer F, Richardson R, Thombs BD, Williams B, McMillan D (2016). Identifying depression with the PHQ-2: a diagnostic meta-analysis. J Affect Disord.

[54] Staples LG, Dear BF, Gandy M, Fogliati V, Fogliati R, Karin E, Nielssen O, Titov N (2019). Psychometric properties and clinical utility of brief measures of depression, anxiety, and general distress: the PHQ-2, GAD-2, and K-6. Gen Hosp Psychiatry.

[55] Chen J, Chen Z (2008). Extended Bayesian information criteria for model selection with large model spaces. Biometrika.

[56] Epskamp S, Fried EI (2018). A tutorial on regularized partial correlation networks. Psychol Methods.

[57] Hastie T, Friedman JH, Tibshirani R (2017). The Elements of Statistical Learning: Data Mining, Inference, and Prediction. 2nd edition.

[58] Dablander F, Ryan O, Haslbeck JM (2020). Choosing between AR(1) and VAR(1) models in typical psychological applications. PLoS One.

[59] Kirtley OJ, Lafit G, Achterhof R, Hiekkaranta AP, Myin-Germeys I (2021). Making the black box transparent: a template and tutorial for registration of studies using experience-sampling methods. Adv Methods Pract Psychol Sci.

[60] Siepe BS, Sander C, Schultze M, Kliem A, Ludwig S, Hegerl U, de Paredes HR Supplementary materials for temporal dynamics of depressive symptomatology: an idiographic time series analysis applying network models to patients with depressive disorders. PsyArXiv.

[61] First MB, Gibbon M, Hilsenroth MJ, Segal DL, Hersen M (2004). The structured clinical interview for DSM-IV Axis I Disorders (SCID-I) and the structured clinical interview for DSM-IV Axis II Disorders (SCID-II). Comprehensive Handbook of Psychological Assessment. Volume 2.

[62] Wittchen HU, Zaudig M, Fydrich T (1997). Strukturiertes Klinisches Interview Für DSM-IV (SKID): Achse I Und II.

[63] Rush AJ, Gullion CM, Basco MR, Jarrett RB, Trivedi MH (1996). The Inventory of Depressive Symptomatology (IDS): psychometric properties. Psychol Med.

[64] Kroenke K, Spitzer RL, Williams JB (2003). The patient health questionnaire-2: validity of a two-item depression screener. Med Care.

[65] American Psychiatric Association (2013). Diagnostic and Statistical Manual of Mental Disorders (DSM-5).

[66] R Core Team R: a language and environment for statistical computing. R Foundation for Statistical Computing.

[67] Moritz S, Bartz-Beielstein T (2017). imputeTS: time series missing value imputation in R. R J.

[68] Bringmann LF, Ferrer E, Hamaker EL, Borsboom D, Tuerlinckx F (2018). Modeling nonstationary emotion dynamics in dyads using a time-varying vector-autoregressive model. Multivariate Behav Res.

[69] Chen M, Chow SM, Hammal Z, Messinger DS, Cohn JF (2021). A person- and time-varying vector autoregressive model to capture interactive infant-mother head movement dynamics. Multivariate Behav Res.

[70] Howe E, Bosley HG, Fisher AJ (2020). Idiographic network analysis of discrete mood states prior to treatment. Couns Psychother Res.

[71] Epskamp S, Cramer AOJ, Waldorp LJ, Schmittmann VD, Borsboom D (2012). qgraph: network visualizations of relationships in psychometric data. J Stat Softw.

[72] Ghosh S (2018). Kernel Smoothing: Principles, Methods and Applications.

[73] Tibshirani R (2018). Regression shrinkage and selection via the Lasso. J R Stat Soc Series B Stat Methodol.

[74] Bean CA, Heggeness LF, Kalmbach DA, Ciesla JA (2020). Ruminative inertia and its association with current severity and lifetime course of depression. Clin Psychol Sci.

[75] Scholten S, Rubel J, Glombiewski J, Milde C What time-varying network models based on functional analysis tell us about the course of a patient's problem. OSF.

[76] Nemesure MD, Collins AC, Price GD, Griffin TZ, Pillai A, Nepal S, Heinz MV, Lekkas D, Campbell AT, Jacobson NC (2024). Depressive symptoms as a heterogeneous and constantly evolving dynamical system: idiographic depressive symptom networks of rapid symptom changes among persons with major depressive disorder. J Psychopathol Clin Sci.

[77] Cramer AO, van Borkulo CD, Giltay EJ, van der Maas HL, Kendler KS, Scheffer M, Borsboom D (2016). Major depression as a complex dynamic system. PLoS One.

[78] Robinaugh DJ, Hoekstra RH, Toner ER, Borsboom D (2020). The network approach to psychopathology: a review of the literature 2008-2018 and an agenda for future research. Psychol Med.

[79] Jaso BA, Kraus NI, Heller AS (2022). Identification of careless responding in ecological momentary assessment research: from posthoc analyses to real-time data monitoring. Psychol Methods.

[80] Albers CJ, Bringmann LF (2020). Inspecting gradual and abrupt changes in emotion dynamics with the time-varying change point autoregressive model. Eur J Psychol Assess.

[81] Bringmann LF (2021). Person-specific networks in psychopathology: past, present, and future. Curr Opin Psychol.

[82] Frumkin MR, Piccirillo ML, Beck ED, Grossman JT, Rodebaugh TL (2021). Feasibility and utility of idiographic models in the clinic: a pilot study. Psychother Res.

[83] Burger J, Epskamp S, van der Veen DC, Dablander F, Schoevers RA, Fried EI, Riese H (2022). A clinical PREMISE for personalized models: toward a formal integration of case formulations and statistical networks. J Psychopathol Clin Sci.

[84] Beltz AM, Wright AG, Sprague BN, Molenaar PC (2016). Bridging the nomothetic and idiographic approaches to the analysis of clinical data. Assessment.

[85] Fisher ZF, Chow SM, Molenaar PC, Fredrickson BL, Pipiras V, Gates KM (2022). A square-root second-order extended Kalman filtering approach for estimating smoothly time-varying parameters. Multivariate Behav Res.

[86] Schuurman NK, Houtveen JH, Hamaker EL (2015). Incorporating measurement error in n = 1 psychological autoregressive modeling. Front Psychol.

[87] Fried EI, Cramer AO (2017). Moving forward: challenges and directions for psychopathological network theory and methodology. Perspect Psychol Sci.

[88] Haslbeck JM, Ryan O (2022). Recovering within-person dynamics from psychological time series. Multivariate Behav Res.

[89] Williams DR, Rhemtulla M, Wysocki AC, Rast P (2019). On nonregularized estimation of psychological networks. Multivariate Behav Res.

[90] Williams DR The confidence interval that wasn’t: bootstrapped “confidence intervals” in L1-regularized partial correlation networks. PsyArXiv.

