# Supplementary material for: Time-Varying Network Models for the Temporal Dynamics of Depressive Symptomatology in Patients With Depressive Disorders: Secondary Analysis of Longitudinal Observational Data
Source: JMIR Ment Health. 2024 Apr 18;11:e50136. doi: 10.2196/50136 (PMC11066753; doi:10.2196/50136)
Supplement: Multimedia Appendix 1 [file mental_v11i1e50136_app1.docx]

# Supplement 1: Theoretical Reasons for Variable Inclusion

In this part of the supplement, we lay out theoretical reasons for why rumination, sleep, and social contacts are relevant variables for a daily diary study of depression.

Rumination, which can be roughly defined as repetitively and passively thinking about the reasons, symptoms and consequences of distress [36], is often considered a transdiagnostic feature or risk factor for mental disorders [35]. Individuals diagnosed with depression show higher levels of rumination compared to controls [37] and rumination has repeatedly been linked to higher risks for depression onset [36]. Based on that, patterns of rumination could provide a specific treatment target for psychotherapy, as can be seen in rumination-focused treatment or mindfulness-based therapies [38]. From a patients’ perspective, Klintwall et al. [34] found that rumination was of central importance for a substantial subset of individuals with depression when asked about causal relations between different depressive symptoms. However, a large study using multilevel vector autoregression found no next-day temporal relationship between rumination and depressive symptoms [39], which may partly be explained by heterogeneity between participants and/or that rumination might be associated with depression on a shorter timeframe, i.e. within minutes or hours.

In alignment with the biopsychosocial model and recent calls to expand the network approach beyond symptoms and psychopathological variables [40], this work also includes the amount and the valence of daily social contacts as rated by participants. There is a long line of research into social impairments associated with depression[41]. In general, depression has for example been found to be associated with lower social support in many studies, but aggregated effects seem to be small [42]. While some studies in everyday life find an association of higher depressive symptom levels with the quantity of social interactions [43,44], several other studies only found lower reported quality, not quantity, of social interactions to be associated with depression [44]. Beyond questions about the exact nature of the relationship of social interactions with depression, a large qualitative study about outcomes of depression treatment indicated that social functioning and social isolation in particular do matter to patients [45]. While the overall relationship of depression and social interactions is well-researched and important to patients, much remains to be known about temporal dynamics and interindividual differences. For example, although more social interactions might in general be advantageous, fewer social interactions might sometimes be beneficial for some individuals, which calls for a more nuanced micro-perspective in everyday life [46]. Further, not absolute levels of social isolation, but the tendency to remain in social isolation for a longer time might be predictive of depressive symptoms [46].

Sleep problems, including insomnia and hypersomnia, are assessed as a symptom of depression in some of the most commonly used depression scales [47] and diagnostic criteria. Overall, sleep alterations are present across various diagnostic categories [48]. Epidemiological studies generally show that a majority of patients with depression show symptoms of sleep disturbances [49]. Longitudinal studies indicate that sleep disturbances and depression can stand in a bidirectional relationship, possibly reinforcing each other [50]. Regarding treatment, cognitive behavioral therapy tailored to treat insomnia has shown promise in ameliorating both sleep problems as well as depressive symptoms [51]. In a previous analysis of the present sample, longer self-reported time in bed was associated with higher depressive symptomatology in eleven participants and lower levels of depressive symptoms in five individuals [30], indicating a substantial interindividual heterogeneity.

In sum, sleep, rumination and socialization are all strongly associated with depression and they all are feasible to assess in self-monitoring diary data. As to be expected, there seem to be substantial interindividual differences in the associations of these variables with depression as well as a general shortage of intensive longitudinal assessments. Further, these variables are not only linked to depression, but likely also connected to each other. For example, some evidence suggests that sleep is also negatively impacted by high levels of rumination [52]. Social interactions also stand in a likely bidirectional relationship to rumination, as social isolation may predict more rumination [46] and people who tend to ruminate more may tend to lose social support [36]. Therefore, we chose to model these variables in our analysis using dynamic ideographic networks.

# Supplement 2: Deviations from preregistration

**Table S1**

*Explained deviations from preregistration*

| **Change** | **Explanation** |
| --- | --- |
| Added that participants could keep the study smartphone as compensation for their completion of the study. | This information was obtained from a data collection responsible only after the preregistration was published. |
| Changed the citation for the German translation of the IDS-C from Boden [32] (2018) to Drieling et al. [31] (2007). | Innocent citation error, as Boden [32] (2018) includes multiple validation studies for the German translation of the IDS. |
| Excluded Participant 10 and 19 from the second iteration of bandwidth selection. | Floor and/or ceiling effects prevented estimation with small bandwidths (no variance at certain estimation points). We did not foresee this issue as it did not occur in our idealized simulations before using real data. |
| Portrayed a case with a light increase in symptom levels instead of cases with stronger increases. | Allowed us to incorporate qualitative information about major life events that were not available for other participants. |
| Various changes to specifics of plotting. | Explained in the manuscript: tailor visualizations to individual cases. |
| Implemented one-standard-error-rule as exploratory analysis. | Explained in the manuscript: check for similar-performing solutions to the optimal solution. |
| Implemented variation of estimation points for participant 2. | Checkfor appropriateness of the number of estimation points for a very small bandwidth. |
| Only presented two instead of three case studies. | Moved case study of participant 2 to the supplement to have more space for other participants and to increase the clinical focus of the manuscript. |

# Supplement 3: STEADY Application

In the following Table S2, all daily self-report items can be found in their original German implementation as well as their English translation. Visual analog scales were displayed without interim answer options or tick marks and only returned integer values. Items were presented in the same fixed order in each log. Morning logs were available from 3 am to 3 pm, while evening logs were available from 3 pm to 3 am.

As noted in the manuscript, the first two items were designed to closely resemble items of the PHQ-2. The PHQ-2 assesses the frequency of symptoms in the past 14 days and is intended as a screening instrument for depression that might be suitable for time-limited settings [53]. Test-retest correlations of around .8 across multiple weeks have been reported [54]. These items were reframed to inquire about a single day for this study.

**Table S2**

*Wording of Items in the STEADY Application*

| **Item** | **German** | **English** | **Scale** |
| --- | --- | --- | --- |
| PHQ1 | „Wie oft fühltest du dich heute während des Tages durch die folgenden Beschwerden beeinträchtigt?“  *“Interessens- oder Freudlosigkeit”* | “How often during the day today did you feel impaired by the following problems?”  *“Loss of interest or joylessness”* | 0-10 „never“ – „all the time“ |
| PHQ2 | „Wie oft fühltest du dich heute während des Tages durch die folgenden Beschwerden beeinträchtigt?“ *“Niedergeschlagenheit, Schwermut oder Hoffnungslosigkeit”* | “How often during the day today did you feel impaired by the following problems?”  *“Feeling down, depression or hopelessness”* | 0-10 „never“ – „all the time“ |
| Sleep | 1. „Wann bist du letzte Nacht ins Bett gegangen?“  2. „Wann bist du letzten Morgen aufgestanden?“  3. „Du lagst insgesamt XXX Minuten im Bett, wie lange hast du davon geschlafen?“ | 1. “When did you go to bed last night?”  2. “When did you get up last morning?”  3. “You lay in bed for a total XXX minutes, how much of that did you spend sleeping?”  (with the XXX calculated from the first two questions) | 1. Time (HH:MM)  2. Time (HH:MM)  3. Minutes |
| Rumination | „Wie oft fühltest du dich heute während des Tages durch die folgenden Beschwerden beeinträchtigt?“  *„Grübeln“* | “How often during the day today did you feel impaired by the following problems?”  *“Rumination”* | 0-10 |
| Social  Quantity | „Wie viele Sozialkontakte hattest du heute?“ | “How many social contacts did you have today?” | 0-100 “none” – “many” |
| Social  Quantity | “Wie empfandest du diese Sozialkontake?“ | “How did you feel about the social contacts?” | 0-100  “unpleasant”- “pleasant” |

*Note.* English wording of questions was translated by the first author. All questions in the app were provided in German. Only the third sleep question was used for analyses.

**Image S1**

*Screenshots of the STEADY application*


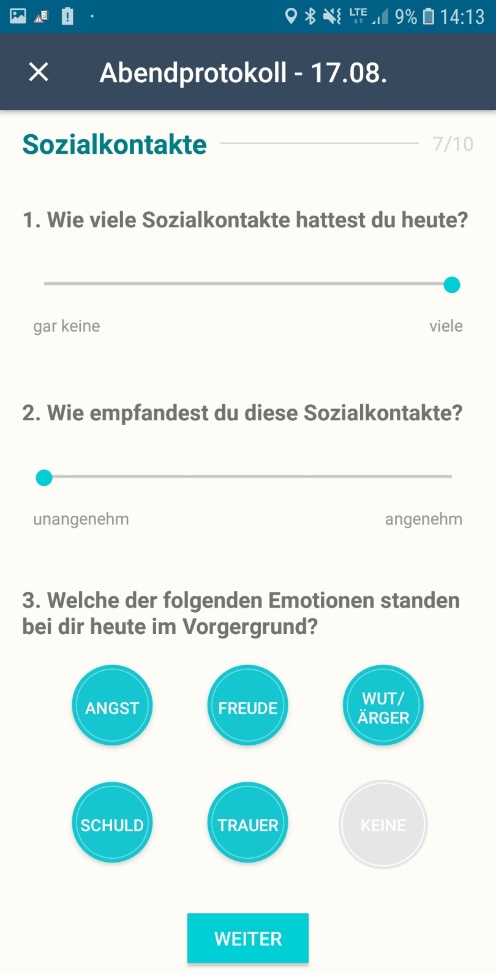

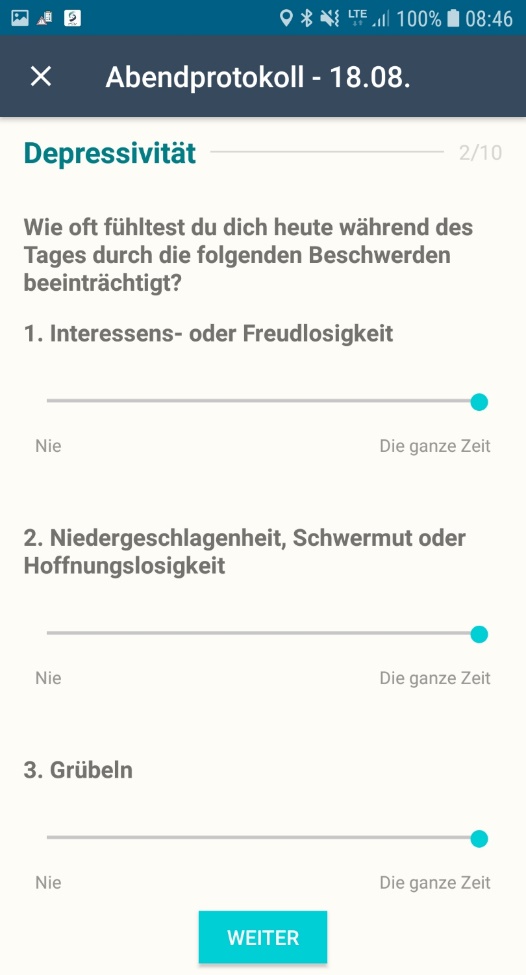

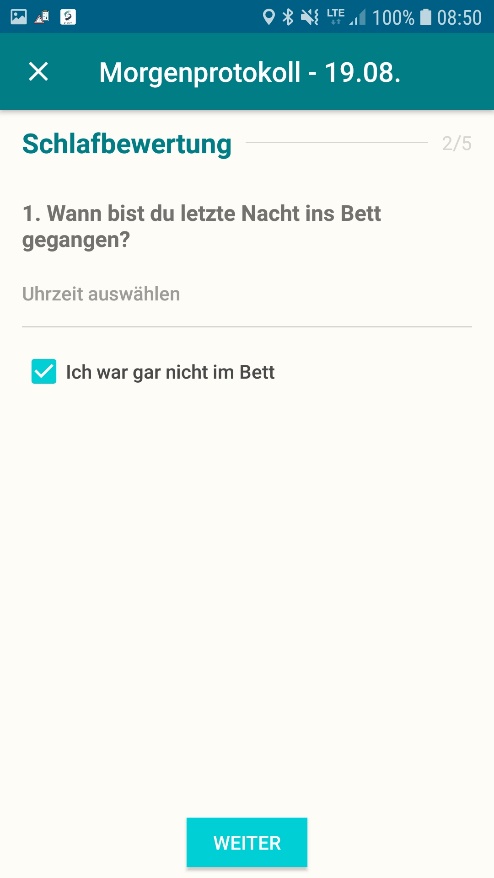


# Supplement 4: Implementation of Time-Varying Vector Autoregression

The time-varying vector autoregressive method uses kernel-smoothing to estimate multiple models pre individual. Kernel-smoothing is a flexible approach to account for nonlinearity in data, including variation over time (see Altman, 1992, for a concise introduction). Instead of assuming stationarity across the whole time series, we assumed local stationarity at different points over time which requires that the model is smoothly dependent on time, thus showing no sudden jumps in parameter values. Haslbeck et al. [25] have shown that even if sudden jumps in parameter values occur, time-varying VAR models may still regularly outperform stationary models.

We used *lasso* regularization to estimate models. The regularization parameter λ controls the extent of the shrinkage. We chose λ based on the Extended Bayesian Information Criterion (EBIC) [55], which has shown a desirable performance in selecting appropriate network models [56]. The EBIC is controlled by setting the hyperparameter γ to an appropriate value between 0 and 1, where higher values imply a preference for parsimony [56]. As in Mansueto et al. [33], γ was set to 0 to achieve a higher sensitivity.

Regarding stability of estimates, we used a block bootstrap scheme in which data were split into 20 blocks with equal length, from which data were resampled in 1000 bootstrap samples. The blocks were then reattached into a new bootstrapped time series. A time-varying VAR model was then fitted to each bootstrap sample, resulting in a distribution of parameter estimates. Bootstrapped sampling distributions of regularized estimates should not be used to perform significance testing to test if an estimate is different from zero [56] as the lasso biases estimates toward zero.

As noted in the manuscript, we tested time series for stationarity. First, we fitted a stationary VAR model to the whole time series. Then, we simulated 100 time series based on the parameters obtained in the first step. On each of these datasets, we then again fitted a time-varying VAR model and computed the overall RMSE of each of these models. The 100 RMSE values then served as the sampling distribution under H_0_ to which we could compare the empirical RMSE for the actual time-varying VAR model of an individual. This empirical RMSE served as test statistic and was compared against the 5%-quantile of the sampling distribution. If the test statistic was smaller than this quantile, we rejected H_0_

# Supplement 5: Bandwidth Selection

As described in the manuscript, we compare candidate bandwidths in a time-stratified five-fold cross-validation scheme. We generated a bandwidth sequence of ten equally spaced values between 0.01 and 1. For each of these bandwidths, the time series was split up into five test sets, where each of the latter contained $\frac{n}{5}$equally spaced timepoints across the whole time series. Time-varying VAR models with the given bandwidth were then fitted on the data, leaving out the timepoints in the test set. Using the resulting model, a prediction error for all six variables was computed for the test set. The bandwidth minimizing the Root Mean Squared Error (RMSE) over all variables in the test sets was then selected. If the highest or lowest bandwidth were selected, we performed an additional iteration of the selection procedure, as suggested in [29]. For participants with a bandwidth of 0.01, we used a second sequence of ten equally spaced values between 0.001 and 0.009; for participants with a bandwidth of 1, we used a second sequence of ten equally spaced values between 1.1 and 2.

For 7 out of 20 participants, the smallest bandwidth (0.01) returned the lowest MSE in the initial bandwidth selection process and was therefore selected as the optimal bandwidth. The second iteration of bandwidth selection for the participants with either the highest or lowest bandwidth led to only one adjustment of the optimal bandwidth from 0.01 to 0.009 (for participant 2). Figure S1 illustrates the practical differences between a relatively small,

medium, and large bandwidth at estimation point 10 of 20. From the figure, it is evident that the difference between the smallest bandwidth in our sample (0.009) compared to a still relatively small bandwidth of 0.12 is already strongly noticeable. A bandwidth of 0.009 leads to a highly localized estimation of parameters, whereas the largest bandwidth of 1 leads to an almost uniform weighting of all points and thus to stable results that are similar to the model assuming stationarity. A small bandwidth thus implies fast changes in the associations between the included variables.

**Figure S1**

*Comparison of Three Different Bandwidths*


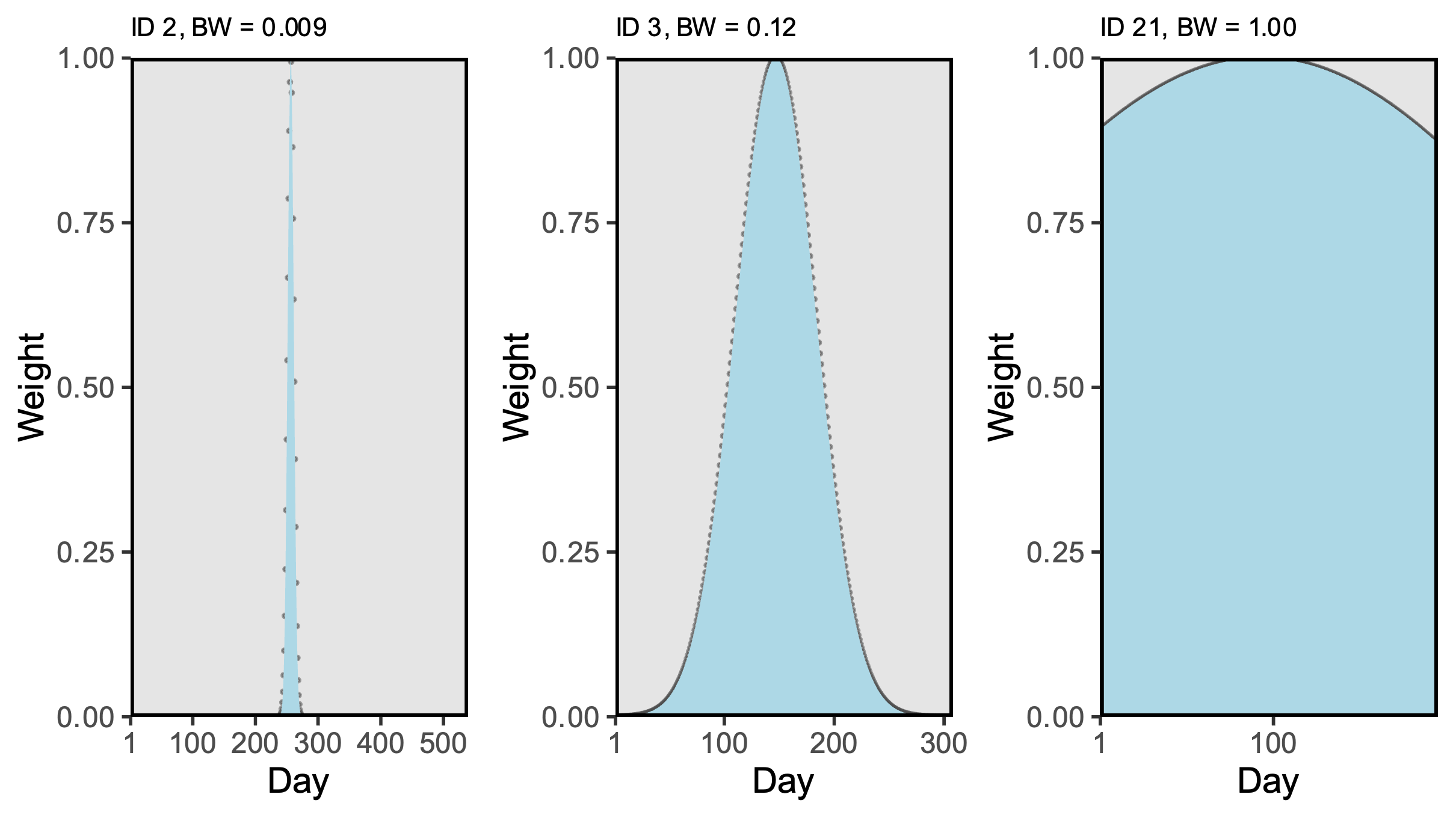


*Note.* The tenth estimation point chosen here is not exactly in the middle of a time series, but rather at around the 47^th^ quantile. For all bandwidths, the weight at the estimation point itself is equal to 1. The weights that were assigned to each timepoint are shown on the y-axis. The area under the curve in blue indicates the amount of information that was used relative to the total amount of information available, which can be visualized as the area of the full black rectangle, amounting to the blue and grey area [29]. All information would be used if every observation would be given an equal weight of 1.

The area under the curve must be interpreted relative to the size of the respective time series, as the same bandwidth includes more information per estimation point the more observations a time series includes. The effective sample size used at an estimation point relative to the whole sample can be obtained by summing all weights at this estimation point [29]. For example, if every observation in a time series of length *n* = 100 would be weighted equally with weight 1, the effective sample size at any estimation point would be 100. In the present example and at the exemplary estimation points, models of participants 2, 3 and 21 used an effective sample size of 12.1, 92.0 and 185.1, respectively.

Judging from the choice of the bandwidth based on the minimum error over the test sets alone, it is unclear if the selected bandwidth performs noticeably better compared to other (larger) bandwidths. Upon visual inspection of the prediction errors of different bandwidths, we noticed that for some participants the RMSE of multiple bandwidths seemed to be nearly identical. It can be argued that when several models perform almost equally, there may be a benefit in selecting more parsimonious/conservative models with a larger bandwidth. In the case of cross-validation, this preference of parsimony is often reflected in the application of the popular *one-standard-error-rule*. According to this heuristic, one selects the most parsimonious model that is within the range of one standard error of the best model [57]. We implemented the rule within an exploratory analysis that was not preregistered and investigated how its implementation would change the choice of bandwidth.

# Supplement 6: One-Standard-Error Rule

The bandwidth selection procedure that we used led to the selection of many small bandwidths, which in turn led to the estimation of highly localized models that may overfit the data. There are several potential remedies for this problem. First, it could be useful to use a more fine-grained bandwidth sequence, especially for lower bandwidths, as the difference between the smallest bandwidths of 0.01 and 0.12 in the initial sequence is already stark. Second, it would be possible to implement a specific lower threshold for bandwidth selection which could be based on the length of the time series and would prevent models that are too flexible to variation over time, therefore facilitating interpretation. The choice of an appropriate threshold would, however, be nontrivial and entails the risk of worsening the performance of the model by decreasing the sensitivity to variation over time. Third, we tested the one-standard-error-rule in exploratory analyses with the goal of erring towards parsimony while still choosing a model that should theoretically not perform much worse than the best model. To do so, we computed cross-validation standard errors by calculating average errors for each of the *k = 5* folds, calculating the standard deviation for the average errors and dividing said standard deviation by the square root of *k*, or, in formula notation:

$SE(RMSE) =\frac{SD({RMSE}_{1}, . . . {,RMSE}_{k})}{\sqrt{k}}$ ,

where *RMSE_k_* is the RMSE of the *k*-th fold of cross-validation.

Since the one-standard-error-rule is just a heuristic, its performance and suitability depend on the data at hand, as [58] have shown in the context of choosing between a simpler autoregressive and a more complex vector autoregressive model. Transferring the gist of their results to our application, it is possible that the one-standard-error-rule for bandwidth selection might perform worse than our initial rule of choosing the bandwidth with the smallest prediction error, especially for a larger sample size or if the true parameters indeed vary highly over time.

Figure S2 illustrates the case of participant 11 where several bandwidths led to similar cross validation errors. Here, a relatively small bandwidth 0.12 was initially chosen, implying a relatively large flexibility to variation over time. However, it is unclear if this bandwidth performs noticeably better than a larger bandwidths, which is why we implemented the one-standard-error rule as explained in the manuscript. The blue region indicates the area within +1 standard error of the selected bandwidth. In this case, the application of the one-standard-error-rule would have led to a selection of the second largest bandwidth of 0.89.

**Figure S2**

*Bandwidth Selection with One-Standard-Error Rule for ID 11*


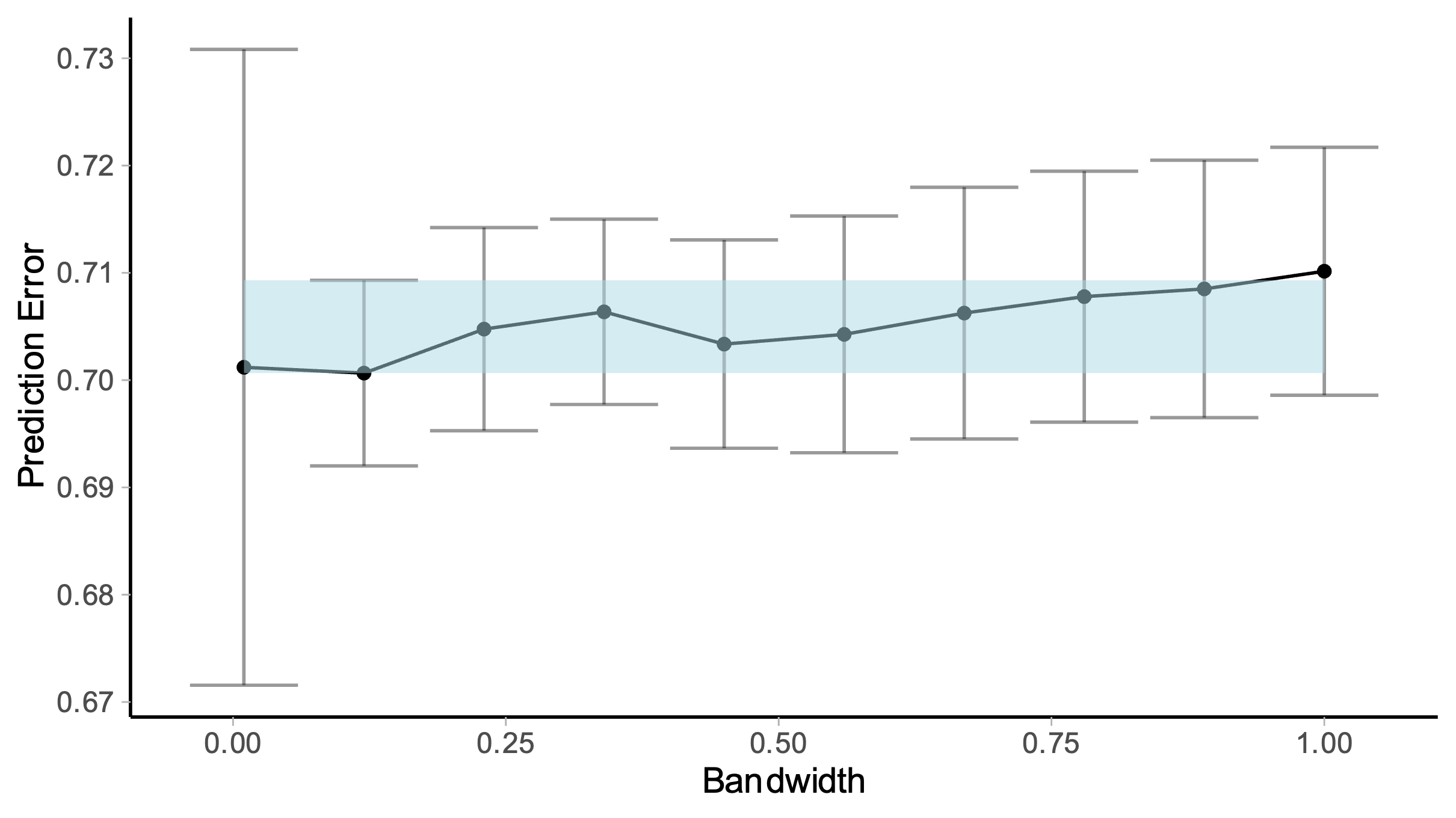


*Note.* Vertical bars depict the interval ± 1 SE around the point estimate.

The following

Table S3 presents the originally chosen bandwidth and the bandwidth that was chosen under the application of the one-standard-error rule. We did not compare the cross-validation errors of a bandwidth of 1 to even larger bandwidths as we were mainly interested in the effect of the rule on smaller bandwidths.

**Table S3**

*Comparison of Original Bandwidth Selection to One-Standard-Error-Rule*

| ID | BW_Original_ | BW_1SER_ |
| --- | --- | --- |
| 1 | 0.45 | 1.00 |
| 2 | 0.009 | 0.01 |
| 3 | 0.12 | 0.12 |
| 4 | 0.12 | 0.23 |
| 5 | 0.12 | 0.34 |
| 6 | 0.12 | 1.00 |
| 7 | 0.23 | 1.00 |
| 8 | 0.01 | 0.01 |
| 9 | 0.01 | 0.01 |
| 10 | 0.01 | 0.01 |
| 11 | 0.12 | 0.89 |
| 13 | 0.12 | 0.23 |
| 15 | 0.01 | 0.01 |
| 16 | 0.01 | 0.01 |
| 17 | 0.12 | 0.12 |
| 18 | 0.23 | 0.45 |
| 19 | 0.01 | 0.12 |
| 20 | 0.12 | 1.00 |
| 21 | 1.00 | 1.00 |
| 22 | 0.12 | 0.12 |

# Supplement 7: Predictability

The following plot illustrates the nodewise *R²* value for each individual model for all participants at all estimation points. Note that this plot contains a slightly different information compared to the average *R²* in the manuscript: In this plot, we only show *R²* values for individual models at a single estimation point averaged over all variables, while the average *R²* in the manuscript computes the *R*² across all time points averaged over all variables.

**Figure S3**

Average Predictability for all Participants
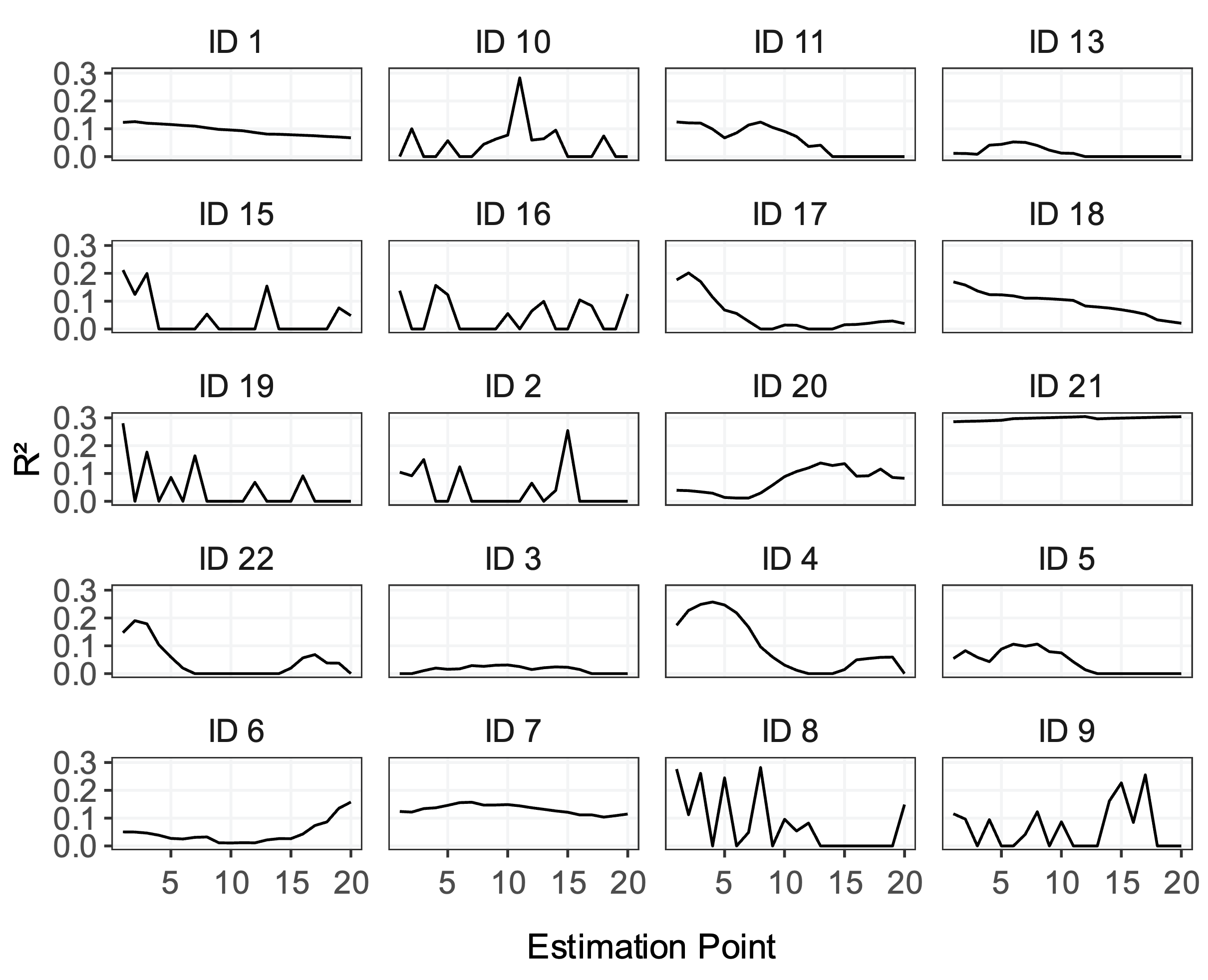


# Supplement 8: Stationarity Tests

As described in the manuscript, we performed a stationarity test as proposed in [25] to test the null hypothesis that the process was stationary. First, we fitted a stationary VAR model to the whole time series of an individual. Then, we simulated 100 time series based on the parameters obtained in the first step. On each dataset, we then fitted a time-varying VAR model and computed the overall RMSE of each of these models. The 100 RMSE values then served as the sampling distribution under the H_0_, to which we could compare the empirical RMSE for the actual time-varying VAR model of an individual. This empirical RMSE served as the test statistic and was compared against the 5%-quantile of the sampling distribution. If the test statistic was smaller than this quantile, we rejected H_0_. The following Table S4 contains the empirical error of the actual model, the 5%-quantile of the sampling distribution, the minimum value of all 100 errors in the sampling distribution and the difference of the empirical error to the mean of the sampling distribution for each participant in the sample.

**Table S4**

*Results of Stationarity Tests*

| ID | Empirical Error | 5%-Quantile Sampling |  | Sampling Minimum | Difference to Mean |
| --- | --- | --- | --- | --- | --- |
| 1 | 0.9460 | 0.9633 |  | 0.9575 | 0.0270 |
| 2 | 0.8483 | 0.9461 |  | 0.9357 | 0.1125 |
| 3 | 0.9720 | 0.9836 |  | 0.9767 | 0.0171 |
| 4 | 0.7677 | 0.8345 |  | 0.8043 | 0.0990 |
| 5 | 0.9273 | 0.9511 |  | 0.9448 | 0.0386 |
| 6 | 0.9368 | 0.9678 |  | 0.9572 | 0.0402 |
| 7 | 0.9148 | 0.9352 |  | 0.9260 | 0.0328 |
| 8 | 0.7413 | 0.8760 |  | 0.8687 | 0.1635 |
| 9 | 0.7135 | 0.8437 |  | 0.8300 | 0.1542 |
| 10 | 0.9162 | 0.9333 |  | 0.9295 | 0.0308 |
| 11 | 0.8688 | 0.9228 |  | 0.9127 | 0.0742 |
| 13 | 0.9042 | 0.9421 |  | 0.9373 | 0.0530 |
| 15 | 0.7982 | 0.8940 |  | 0.8860 | 0.1149 |
| 16 | 0.8175 | 0.9106 |  | 0.8948 | 0.1096 |
| 17 | 0.8508 | 0.9568 |  | 0.9410 | 0.1184 |
| 18 | 0.9282 | 0.9458 |  | 0.9385 | 0.0342 |
| 19 | 0.7980 | 0.9061 |  | 0.8965 | 0.1274 |
| 20 | 0.9390 | 0.9705 |  | 0.9685 | 0.0423 |
| 21 | 0.8280 | 0.8771 |  | 0.8683 | 0.0707 |
| 22 | 0.8820 | 0.9544 |  | 0.9453 | 0.0851 |

# Supplement 9: Missing Data

Table S5 shows the percentage of missingness on each variable for each participant.

**Table S5**

*Missing Data Summary*

| ID | PHQ1 | PHQ2 | Sleep | Rumination | Social Quantity | Social Quality |
| --- | --- | --- | --- | --- | --- | --- |
| 1 | 11.90 | 11.90 | 17.04 | 11.90 | 11.90 | 11.90 |
| 2 | 5.01 | 5.01 | 4.64 | 5.01 | 5.01 | 5.01 |
| 3 | 3.90 | 3.90 | 2.92 | 3.90 | 3.90 | 3.90 |
| 4 | 14.71 | 14.71 | 13.73 | 14.71 | 14.71 | 14.71 |
| 5 | 3.90 | 3.90 | 3.41 | 3.90 | 3.90 | 3.90 |
| 6 | 2.33 | 2.33 | 1.99 | 2.33 | 2.33 | 2.33 |
| 7 | 7.12 | 7.12 | 5.50 | 7.12 | 7.12 | 7.12 |
| 8 | 1.77 | 1.77 | 1.77 | 1.77 | 1.77 | 1.77 |
| 9 | 3.17 | 3.17 | 1.59 | 3.17 | 3.17 | 3.17 |
| 10 | 4.43 | 4.43 | 3.16 | 4.43 | 4.43 | 4.43 |
| 11 | 0.98 | 0.98 | 0.98 | 0.98 | 0.98 | 0.98 |
| 13 | 1.65 | 1.65 | 2.31 | 1.65 | 1.65 | 1.65 |
| 15 | 8.37 | 8.37 | 6.46 | 8.37 | 8.37 | 8.37 |
| 16 | 3.65 | 3.65 | 3.65 | 3.65 | 3.65 | 3.65 |
| 17 | 2.60 | 2.60 | 4.55 | 2.60 | 2.60 | 2.60 |
| 18 | 18.18 | 18.18 | 26.77 | 18.18 | 18.18 | 18.18 |
| 19 | 2.64 | 2.64 | 2.97 | 2.64 | 2.64 | 2.64 |
| 20 | 4.98 | 4.98 | 2.99 | 4.98 | 4.98 | 4.98 |
| 21 | 5.67 | 5.67 | 14.95 | 5.67 | 5.67 | 5.67 |
| 22 | 1.32 | 1.32 | 1.66 | 1.32 | 1.32 | 1.32 |

*Note.* Missing data per item in percentages. The peculiar structure of 5 items with the same amount of missingness while sleep shows a different amount of missingness arises from the division into evening and morning protocols.

# Supplement 10: Case Study Participant 2

Participant 2 was a German woman in her fifties who worked full time, took multiple antidepressants and had previously been in psychotherapeutic treatment. For her time series with a length of 539 days, by far the longest data collection span in the whole sample, a bandwidth of 0.009 was selected. Her case is presented due to the length of her time series and the large extent of variation over time. In Figure S2, we visualize network models at all estimation points where any edge was nonzero, leaving out 14 estimation points without any edges. As these networks display all estimated nonzero effects, we do not present time-varying effects separately. Some qualitative information about her life during data collection was also available. Shortly after the ninth estimation point, participant 2 complained about increased stress and longer working hours. Between estimation points 12 and 13, a friend of the participant received a severe medical diagnosis. Networks after both these adverse events were estimated empty, as they were at many other points.

Due to the small bandwidth, the default number of 20 estimation points might not have been adequate to capture all time- varying effects. To investigate this, we modelled the number of estimation points for participant 2 up until fitting a model at every 539 timepoints. In this explorative analysis, we observed substantial changes when including more estimation points, indicating that our initial setup was too coarse. While individual parameters differed, the general pattern of results remained roughly the same, insofar as effects varied strongly and rapidly over time and as many networks were empty. We present these results as an animated plot in the electronic supplement. As evident in Figure S4, edges at the six included estimation points varied widely over time, with rumination having outgoing edges at three estimation points. Besides the positive cross-lagged effect from rumination on feeling down at estimation points 3 and 15, no edge was present more than once. If edges were present, their weight was generally large, with a median absolute nonzero weight of 0.45. For long intervals across the time series, no effect was present in the networks. The average R²-value over all estimation points and variables was 0.272.

**Figure S4**

*Nonempty Networks for Participant 2 at Estimation Points 1, 2, 3, 12, 14, and 15*


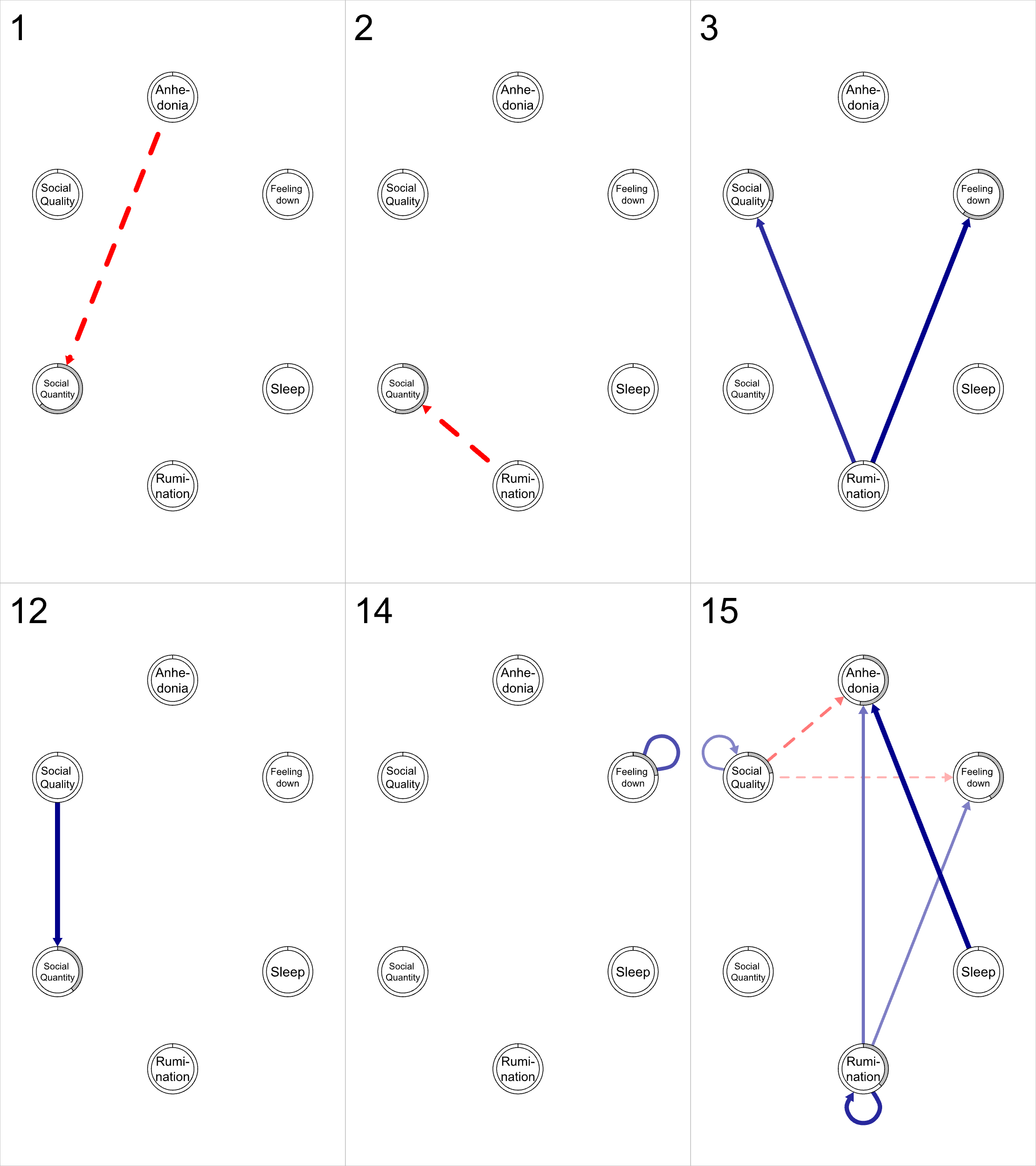


*Note.* Arrows (*edges*) from one variable (*node*) to another represent lag-1 temporal associations. Arrows from a variable to itself represent lag-1 autocorrelations. Blue/red arrows indicate positive/negative relationships, respectively. The grey pie chart around a variable indicates the *R*²-value of that variable at a given estimation point.
